# HOW MISUSE OF ANTIMICROBIAL AGENTS IS EXACERBATING THE CHALLENGES FACING SOMALIA’S PUBLIC HEALTH

**DOI:** 10.21010/Ajid.v16i2S.4

**Published:** 2022-08-17

**Authors:** Moussa Ayan Aden, Garba Bashiru

**Affiliations:** † Institute for Medical Research, SIMAD University, Mogadishu, Somalia; # Department of Veterinary Public Health and Preventive Medicine, Faculty of Veterinary Medicine, Usmanu Danfodiyo University, Sokoto, Nigeria

**Keywords:** Antimicrobial Resistance, Public Health, Emerging Diseases, Somalia, Antimicrobial Stewardship

## Abstract

**Background:**

In contrast to most developed countries, antimicrobial resistance (AMR) has continued to be a serious challenge to public health in the majority of resource-limited countries in Africa.

**Materials and method:**

A comprehensive review of all available literature reporting on antimicrobial resistance patterns, antimicrobial drug usage in both human and animals, as well as national AMR regulations in Somalia was undertaken.

**Results:**

The review observed that successful AMR control and surveillance among resource-poor nations are affected by a lack of infrastructural and institutional capacities, poor investment in human and material resources, as well as non-adherence to available policies. The humanitarian crisis affecting Somalia has persisted for too long, leading to loss of lives, productivity and dilapidation of public health infrastructures. Somalia like most countries has adopted the One Health approach in developing their soon-to-be gazetted National Action Plan on AMR, which covers both human health, animal health and the environment. Although there are many other similar policy documents and guidelines regulating the usage and administration of antimicrobials in the country, evidence of the implementation indicates there is still a need for more effort.

**Conclusion:**

AMR constitute a significant public health problem in Somali, and there is urgent need for gazetting and enforcement of the newly developed national policy. In addition, there is also the need for collaboration with the major stakeholders to develop workable solutions to combat the hazards posed by AMR in the country.

## Introduction

The emergence of antimicrobial-resistant microbes has gained popularity in recent years and has been adjudged one of the major public health challenges facing the world. The development of resistance among infectious disease pathogens is driven by the irresponsible usage of antimicrobial agents in human healthcare settings as well as in veterinary medicine (Salihu *et al.*, 2013; Prestinaci *et al.*, 2015; Abdulhaleem *et al.*, 2019; Hall *et al.*, 2020; Gaddafi *et al.*, 2021). On the other hand, genetic evolution and transfer of mobile genetic elements via horizontal gene transfer are also some of the major factors playing roles in the continued emergence of antimicrobial-resistant pathogens (Liu *et al.*, 2016; Spohn *et al.*, 2019). In addition to the health hazards AMR poses, reports have also shown severe economic consequences including the financial burden on families due to long hospitalization, and also reduced productivity in the livestock industry (Aslam *et al.*, 2018; Garba *et al.*, 2019).

The situation is not different in Somalia compared to the global trends. However, it is difficult to discuss Somalia without looking at the impact of its long-standing unrest as a result of political conflicts and insurgency which led to the displacement of its citizens and the dilapidation of health services and infrastructure. Reports indicate that about 19.3% of the country’s population lives in rural areas while the remaining 80.7% reside in urban towns and cities (Linard *et al.*, 2010). The conflicts and the resultant poor healthcare delivery had also affected the human population in terms of public health. These include malnutrition, poor personal and environmental hygiene, inadequate potable water, low immunization rates and high disease burden as well as poor institutional capacity and financing. Other important challenges the country is experiencing are the lack of skilled health workforce while the few are poorly remunerated and hence not fully motivated, in addition to uneven access to essential health services (WHO 2017). This continued deterioration of national security poses a significant threat to attaining public health sufficiency in accordance with the WHO recommendations.

The proliferation of AMR pathogens in Somalia portends danger since the usually effective drugs have lost their efficacy, and hence cannot treat ailments they are used for. Among the many groups of infectious disease pathogens that have shown the potential to become resistant to the available drugs, the development of resistance by bacterial organisms is of significant concern (Gajdács *et al.*, 2021). The WHO is advocating for substitution with last-line drugs (carbapenems and polymyxins), unfortunately, these drugs are not readily available in Somalia.

### Overview of Somalia’s Public health challenges

Official statistics on the burden of diseases (infectious, non-communicable, zoonotic) are scanty. Nonetheless, according to the WHO documents, the common diseases with the most morbidity and mortality include: cholera, tuberculosis, malaria, and measles (Holmes *et al.*, 2017). Other notable zoonotic and emerging diseases are the Coronavirus group (SARS, MERS-CoV), and SARS-CoV-2, food-borne infections like brucellosis, and toxoplasmosis, as well as leishmaniosis mostly affecting rural settings with poor hygiene and sanitation ( Garba *et al.*, 2018; Neela *et al.*, 2019; Supakul *et al.*, 2019; Nur *et al.*, 2022; Adam *et al.*, 2022;).

In recent years, the Ministry of Health of Somalia has recognised antimicrobial resistance as one of the biggest public health challenges in Somalia. This global public health problem is driven by irresponsible self-prescription and misuse of antimicrobials which has resulted in the increasing prevalence of resistant pathogens thereby weakening the ability to treat common infections (Odenwald *et al.*, 2007; Mohamed *et al.*, 2020; Moussa *et al.*, 2021). Other non-diseased public health problems affecting Somalia are malnutrition (19.5-37%) caused by long periods of drought and famine and food insecurity. Another important problem affecting Somalia is limited access to basic healthcare services. This limitation is being exacerbated by insecurity in some regions of the country, thereby contributing to the persistence and spread of diseases.

### Status of AMR and its drivers in Somalia

Antimicrobial resistance (AMR) is said to have occurred when drugs that are usually effective against an organism lost their efficacy. What follows this outcome is detrimental and can include treatment failures, increased morbidity with severe outcomes as well as deaths. The list is in-exhaustive, hence the popularity the problem is gaining globally. According to the European Antimicrobial Resistance Surveillance Network (EARSNet), over 670,000 infections with resistant bacteria are recorded in Europe annually, among which 5% (33,000) die from the direct consequence of these infections (ECDC, 2019).

The situation in Somalia is even direr due to the absence of quality healthcare services and health infrastructure occasioned by the ongoing conflict. Notable among the AMR bacteria causing serious concern to the Somali government is *Mycobacterium tuberculosis*. During the year 2013, about 3300 tuberculosis (TB) cases were detected in the first quarter of 2013 according to the WHO-Somalia Situation Report-2013 (WHO 2013). Analysis of this data by a TB centre in Garowe discovered that 5.2% of these cases are new, while 41% are previously treated TB cases which have turned out to exhibit Multi-Drug Resistant (MDR) properties (WHO 2013). As a result of this finding and the emergence of the anti-TB drug resistance strain of *Mycobacterium*, the WHO has engaged with major stakeholders in Somalia to initiate MDR-TB management in Somalia. Similarly, a recent investigation by Moussa et al. showed that many bacterial pathogens in Somalia have demonstrated varying levels of resistance (Moussa *et al.*, 2021).

Hence, the need for a holistic effort to survey and determine the AMR profile of the common bacterial organisms isolated from healthcare settings.

Reports have shown that irrational use of medicines such as polypharmacy, overuse of antibiotics and injections are some of the major contributors of the development of resistance to infectious disease agents in most countries, especially those countries battling with conflicts and other humanitarian challenges like Somalia, where the government is very weak and is overwhelmed by security challenges such that less attention is given to controlling drug administration and usage among its citizens (Odenwald *et al.*, 2007; Hall *et al.*, 2020;). Investigations in the Galmudug state of Somalia revealed that of 3014 medicines prescribed during a two-month study period, 946 (31.2%) were antibiotics and 889 (29.5%) were analgesics. The study found that the most prescribed antibiotics were amoxicillin, and metronidazole, as well as the anthelmintic albendazole (Hassan, 2021). The author concluded that there is high prevalence of irrational use of drugs in terms of polypharmacy and overuse of antibiotics and poor knowledge of drug prescribers about the rational use of drugs against the existing WHO standard treatment guidelines.

Preliminary surveillance of antimicrobial usage as well as evaluation of knowledge and awareness of medical practitioners in hospitals in Mogadishu by researchers at SIMAD University, Mogadishu, Somalia also indicate that Amoxicillin is the drug with the most resistance among a group of Enterobacteriaceae studied that included *Salmonella*, *E. coli*, *Shigella*, *Streptococcus, Klebsiella*, as well as other common pathogens like *Neisseria*, *Staphylococcus* and *Giardia*.

An assessment of drug utilization also showed, worryingly, poor standards of practice observed amongst medical practitioners working in both private and public health institutions (Bhui & Warfa, 2007; Mohamoud *et al.*, 2017). These assessments also found that over prescription and self-prescription is a common practices, which may account for the high drug consumption rate (Bhui & Warfa, 2007; Mohamoud *et al.*, 2017). Preliminary results regarding the awareness level of medical practitioners with respect to antimicrobial resistance awareness showed that they generally do not lack awareness and they have relevant guidelines including official drug formulary, hence we are assuming negligence as a possible reason why despite knowing the implications, the practice of irresponsible prescription persist.

### National action plan on antimicrobial resistance

In recent years, the Somali government and other local and international health authorities have been actively involved in tackling AMR in the country. In the year 2020, Somalia, with support from the World Health Organisation, jointly formulated a National Action Plan to combat antimicrobial resistance (Bano, 2021). This policy document was formulated after the long-overdue absence of a national regulatory body, in line with the WHO recommendation. The absence of such a regulatory body is believed to be the reason behind the proliferation of numerous uncertified private pharmaceutical suppliers that continue to flood the country with substandard imported medicines which have contributed immensely to the AMR crises in Somalia.

Although the proposed National Action Plan has not been officially gazetted, it identified the frequent misuse and overuse of antimicrobials, particularly among healthcare professionals and those engaged in animal husbandry to be the main drivers in the development of drug-resistant pathogens. This desirable intervention could not have come at a better time than now, especially with the increasing chances of prescription medicines losing their efficacy at treating common infections, which in turn is increasing the risk of disease spread, the severity of illness and mortality.

### AMR Surveillance capacity

Public health laboratories have been playing a significant role in the fight against AMR, by identifying emerging resistance mechanisms as well as responding to disease outbreaks involving resistant pathogens. These laboratories can also help detect emerging threats, thereby preventing the further spread of antimicrobial resistance in addition to strengthening surveillance and management systems (Bunduki *et al.*, 2020). The routine surveillance of AMR organisms has proven to be an important strategy for effective monitoring of outbreaks associated with antimicrobial-resistant pathogens. It usually entails identifying populations most at risk, as well as designing and evaluating suitable intervention strategies. Monitoring of AMR requires standard quality and functional microbiology laboratory. Unfortunately, the importance of clinical microbiology laboratory services has not been fully recognized by the Somali government and indeed its public health institutions. Currently, only a handful of tertiary and secondary care hospitals have standard clinical microbiology laboratories (WHO, 2016).

Following the WHO-IHR evaluation of Somalia’s capacity to monitor and survey AMR, it was discovered that there was no functional multidisciplinary agency required to effectively combat AMR. However, three laboratories located in Hargeisa, Mogadishu, and Bosaso were found to have the facilities and skills to identify AMR pathogens including AMR *Mycobacterium tuberculosis* from human specimens. In addition to these human laboratories, Somalia also has a central Veterinary Laboratory, as well as the National Public Health Reference Laboratory (NPHRL) capable of detecting circulating AMR pathogens. These are grossly insufficient considering the magnitude of health challenges AMR represents globally. Hence, the urgent need for the government to invest in building and enhancing the country’s laboratory capacity, in order to meet up with the challenge. Nonetheless, it is worthy to commend the Somalia government’s efforts for its success in establishing a very good surveillance program for detecting drug resistance to TB, which Somalia is reported to have the highest rate in Africa (Médecins Sans Frontières, 2022; WHO-EMRO, 2022).

### AMR among human and animal populations

The rate at which AMR is developing among humans and animals is disturbing. Antibiotics were generally used for treatment, prophylaxis and as additives in livestock feed to increase productivity. Despite the potential benefits associated with their usage in animal feed, the unregulated use and over-use have led to evolutionary changes and genetic selection of resistant strains, resulting in the emergence of drug resistance in animals (Musawa *et al.*, 2020; Zakaria *et al.*, 2020, 2021, 2022). By the nature of our interaction with animals as food sources and companionship, humans have increasingly become prone to contracting diseases caused by these resistant pathogens (Salihu *et al.*, 2015; Abdulhaleem *et al.*, 2019; Karim *et al.*, 2021;). Unfortunately, infection with these resistant pathogens has come with dire consequences including long hospital stay due to treatment failures as well as other detrimental health problems caused by AMR.

It has therefore become necessary for increased awareness of the judicious use of these agents, followed by the adoption of policies in the country that will promote good antimicrobial stewardship practice in both the human healthcare system and veterinary services. This one health approach is being advocated by the WHO, where provisions that address the prevention and control of AMR pathogens is incorporated into the national programs as seen with the newly established Somalia National Action Plan on AMR.

### Misuse and overuse of antibiotics as drivers of AMR in Somalia

Consumption or administration of drugs when not required or wrongly prescribed are common in human healthcare and veterinary services. This attitude has been adjudged the major contributor driving the rapid development of resistance among infectious disease pathogens. The rate at which AMR is emerging globally is alarming. More troubling is the emergence of novel resistance mechanisms that are making successful treatment of common illnesses to become impossible (Achi *et al.*, 2020; Jibril *et al.*, 2020).

The world has witnessed situations where infectious disease agents like *Escherichia coli, Klebsiella*, and *Staphylococcus aureus* that are easily treated become untreatable. This situation is even worse in developing and under-developed countries where the sale of drugs is largely unregulated. Also, in these countries such as Somalia, many of the usually effective medications are often over-prescribed due to a lack of alternatives (Odenwald *et al.*, 2007). Ironically, Somalia has a policy document termed “Somalia standard treatment guidelines and training manual on rational management and use of medicines at the primary health care level” that stipulated guidelines and provisions that will ensure the prudent use of antimicrobials during treatments in the country. However, these provisions are not being adhered to and no effort is being made to enforce their application (Odenwald *et al.*, 2007). This may consequently lead to a serious outcome where common infections and minor injuries can again kill (post-antibiotic era).

### Antimicrobial stewardship activities

As proclaimed by Dr Mamunur Rahman Malik (Head of Mission and WHO Representative for Somalia), “Education is a key component in driving action” against antimicrobial resistance. Raising awareness and education has always been one of the vital means of controlling AMR globally (Manyi-Loh *et al.*, 2018). The WHO recommends that, improving awareness of the hazards of AMR and the advocacy of interventional behavioural change, using education and effective communication strategies as tools, are critical steps that will help stem the tide in AMR development.

“Antimicrobial stewardship” has become a popular phrase when discussing AMR. It is a strategy adopted by the WHO and most developed countries to promote the responsible administration and sale of antimicrobial agents, including guidance on selecting the most effective drug, dosage and period of administration (Al-Tawfiq *et al.*, 2017).

The successful prevention and control of AMR in Somalia will require all clinicians both in public and private health institutions as well as veterinarians and livestock farmers imbibe the spirit of good stewards of antimicrobials by acting responsibly while prescribing drugs for either treatment of diseases or during animal feed preparations. These key stakeholders will also need to assist in educating their clients on the prudent use of drugs. Policy documents to assist them in achieving these objectives are already available including the National Medicines Policy, and the National Standard Treatment Guidelines (revised in 2016). Despite the availability of these policies, the government need to establish a law that makes the prescription of antibiotics mandatory. This will put a stop to the sale of drugs by pharmacies without prescriptions. Secondly, non-availability in the country of some antimicrobials listed in the Essential Medicines List tends to limit the choice of prescription drugs leading to over-prescription of the available drugs that will eventually result in the development of resistance by the disease agents. Finally, it has become evident that AMR awareness among the human health and animal sectors is not sufficient, hence, there is a need for continued training and education of all relevant stakeholders on the knowledge of AMR and its resultant health hazards.

### Concluding remarks

Although antimicrobial resistance is a global public health problem, the incidence can be reduced considerably in Somalia when forces are joined to spread awareness and stop the upward growth of resistant pathogens. This can be achieved when all stakeholders including policymakers, professionals in the health care industry, as well as community members, and business personnel engaged in pharmaceutical drug distribution and supply participate in promoting good practices and spreading awareness about the proper use and sale of medicines. The government of Somalia should also make efforts at strengthening regional and country-wide laboratory networks by creating avenues that will improve staff capacity in diagnosis and identification of resistant trends in the country. Antimicrobial Sensitivity Tests protocols should be harmonized across the country by enforcing laboratories or institutions to develop and adhere to appropriate biosafety and biosecurity guidelines. In addition, the building of institutional and staff capacity will also be very helpful. Stakeholders should seize the opportunities of AMR training sessions offered by many international organisations and NGOs in the country to improve their knowledge and skills for better services.

Furthermore, the country should look into the possibility of enhancing national disease surveillance using modern electronic data platforms. Currently, only AMR-TB and surveillance are being undertaken. There is,, a need to increase these surveillance efforts to all AMR pathogens within the human and animal health spaces. This can be achieved by enrolling in the Global Antimicrobial Surveillance System (GLASS) which is a global system that monitors the collection, identification, and dissemination of AMR-related data by countries around the world. Through such collaboration, the Ministry of Health, for instance, can expand existing national TB surveillance networks to include other priority pathogens as earlier suggested.

### Conflict of interest:

The authors declare that there is no conflict of interest associated with this study.

**Abbreviations:**
AMR-: Antimicrobial resistance,
WHO-: World Health Organisation,
EARSNet-: European Antimicrobial Resistance Surveillance Network,
GLASS-: Global Antimicrobial Surveillance System,
TB-: Tuberculosis
MDR-: Multidrug Resistance,
WHO-: IHR-World Health Organisation-International Health Regulation.
